# Corneal remodeling after SMILE for moderate and high myopia: short-term assessment of spatial changes in corneal volume and thickness

**DOI:** 10.1186/s12886-023-03148-0

**Published:** 2023-10-06

**Authors:** Yuanpeng Wu, Ting Shen, Lingtong Tan, Ting He, Qingqing Zheng, Chaoyang Hong

**Affiliations:** 1 School of Public Health, Hangzhou Medical College, Hangzhou, Zhejiang China; 2 Eye Center, the Second Affiliated Hospital, Zhejiang University School of Medicine, Hangzhou, Zhejiang China; 3 Department of Ophthalmology, Zhejiang Provincial People’s Hospital, Affiliated People’s Hospital, Hangzhou Medical College, Hangzhou, Zhejiang China; 4 School of Ophthalmology and Optometry, Wenzhou Medical University, Wenzhou, Zhejiang China

**Keywords:** SMILE, Moderate myopia, High myopia, Corneal remodeling, PTI

## Abstract

**Purpose:**

To evaluate the early corneal remodeling and its influencing factors after Small incision lenticule extraction (SMILE) for moderate and high myopia.

**Methods:**

This was a retrospective study. Pre- and post-operative (1 week and 1, 3, 6 months) corneal volume (CV), mean keratometry (Km), and corneal thickness (CT) were measured by Scheimpflug tomography. CT at the central, thinnest point, and on concentric circles of 2, 4, and 6 mm diameter was recorded to assess corneal thickness spatial profile (CTSP) and percentage of thickness increase (PTI) in the moderate and high myopia groups, and to explore possible influencing factors.

**Results:**

After SMILE, the peripheral CT decreased in the moderate myopia group and central corneal thickness (CCT) increased in the high myopia group at 1 month compared to 1 week (all *P* < 0.05). The CV, Km and CT were significantly increased at 3 months compared to 1 month (all *P* < 0.05), but there was no significant change at 6 months compared to 3 months for both groups (all *P* > 0.05). Patients with high myopia showed greater corneal thickness changes (△CT) and higher PTI than moderate myopia (all *P* < 0.05). Regression analysis revealed that in addition to refraction, peripheral PTI was negatively correlated with CCT in the moderate myopia group (4 mm: *β* = -0.023, *P* = 0.001; 6 mm: *β* = -0.050, *P* < 0.001), as well as in the high myopia group (4 mm: *β* = -0.038, *P* < 0.001; 6 mm: *β* = -0.094, *P* < 0.001). Moreover, peripheral PTI in the moderate myopia group was negatively correlated with age (4 mm: *β* = -0.071, *P* = 0.003; 6 mm: *β* = -0.162, *P* < 0.001).

**Conclusions:**

After SMILE, the CV, Km, and CTSP showed dynamic changes in the early stage, which stabilized after 3 months. Compared to the moderate myopia group, the high myopia group experienced slower corneal stabilization. The change in PTI at 6 months after SMILE may be related to higher preoperative refraction, thinner CCT and younger age.

**Supplementary Information:**

The online version contains supplementary material available at 10.1186/s12886-023-03148-0.

## Introduction

Myopia is the leading cause of visual impairment worldwide currently. The prevalence of myopia is increasing year by year with the popularity of electronic products and the increase of near work. It is predicted that the global myopia population will reach 4.758 billion in 2050, of which high myopia will reach 938 million, accounting for 9.8% of the total world population [1]. With the improvement of people’s living standards and health requirements, more and more patients with moderate and high myopia choose femtosecond laser surgery to correct myopia in order to remove glasses.

As a newer corneal refractive surgery, Small incision lenticule extraction (SMILE) has been widely accepted due to its many advantages such as no corneal flap, minimal invasion, rapid recovery, and fewer complications [2]. By scanning the interlayer of the cornea, the lenticule is prepared and removed, and the thickness and curvature of the cornea are changed to achieve the purpose of removing the glasses. For a long time, the safety, stability, and effectiveness of SMILE have been a continuing concern for clinicians and patients. Studies have shown that changes in corneal volume (CV), curvature, and thickness distribution occur after SMILE for corneal remodeling [3, 4], and these are not only important indicators for evaluating the safety and stability of refractive surgery, but also for effectively monitoring the occurrence of complications such as corneal ectasia or secondary keratoconus [5–8]. Therefore, the stability of CV, corneal thickness (CT) and curvature are essential for patients after SMILE. Compared with patients with low myopia, patients with moderate and high myopia have a higher incidence of corneal instability and iatrogenic corneal ectasia postoperatively [3], so it is more important for them to evaluate the corneal repair and stability after SMILE. However, there are limited studies on corneal remodeling after SMILE in moderate and high myopia, and the spatial distribution of the corneal profile and its specific changes in the process of postoperative remodeling remain to be investigated.

CV, mean keratometry (Km), corneal thickness spatial profile (CTSP), and percentage of thickness increase (PTI) can provide effective information for corneal remodeling postoperatively as quantitative indexes of corneal histology and morphology [9–11]. Therefore, our study aimed to investigate the early corneal remodeling process after SMILE in patients with moderate and high myopia, including the short-term evaluation of CV, CTSP, and PTI, as well as to explore the related factors.

## Methods

### Subjects

In our present retrospective study, A total of thirty patients (60 eyes) with moderate myopia and 30 patients (60 eyes) with high myopia underwent SMILE at the Eye Center of the Second Affiliated Hospital of Zhejiang University from December 2020 to December 2022 were included. The criteria for inclusion were age ≥ 18 years, spherical equivalent refraction (SER) ≥ -3.00D, refractive stability for more than 2 years, central corneal thickness (CCT) > 480 μm, cessation of soft contact lens wear for at least 2 weeks and hard contact lens wear for at least 4 weeks before the evaluation. Patients with systemic diseases, active ocular diseases, a history of ocular surgery or trauma, keratoconus or suspected keratoconus, and psychiatric disorders were excluded. The study protocol was approved by the Ethics Committee of the Eye Center of the Second Affiliated Hospital of Zhejiang University, and the tenets of the Declaration of Helsinki were followed throughout the study.

### Data collection

All Patients were examined preoperatively and at 1 week, 1 month, 3 months and 6 months postoperatively, including computerized optometry and examination of best-corrected visual acuity (BCVA), non-contact intraocular pressure (IOP), slit-lamp microscope and dilated fundus. Corneal parameters were measured using the Pentacam anterior segment analyzer (OCULUS GmbH, Wetzlar, Germany), which was scanned and analyzed in a dark room when the screen showed “OK”, with multiple measurements per eye and the best image selected. Pre- and post-operative CV and Km were collected at different time points, as well as four points were collected at the corneal apex, the thinnest point (TP), and the 45°, 135°, 225° and 315° meridians of the 2, 4, and 6 mm diameter concentric rings, for a total of fourteen points, and calculated the sum of CT values on each ring and its mean value, respectively. Patients were divided into the moderate myopia group (-3.00 ≤ SER < -6.00D) and the high myopia group (SER ≥ -6.00D) according to SER.

### Main outcome indicators

The CV, Km, CTSP, and PTI were evaluated with Pentacam. The CTSP evaluation included measurements of the CCT, the minimum corneal thickness (MCT), and the average corneal thickness on 2, 4, and 6 mm rings, where the central (CTR) and 2 mm diameter ring were defined as the central cornea and the 4, 6 mm diameter rings were regarded as the peripheral cornea [12]. △CV, △Km, and △CT are respectively the changes of CV, Km, and CT at adjacent time points in the same area after SMILE, with positive values being an increase and negative values being a decrease. PTI evaluation was performed using the formula: PTI = (CT@x - MCT) / MCT [13, 14], where x represents the diameter of each ring centered on the TP, including 2, 4, and 6 mm zones.

### Surgical techniques

All SMILE procedures were performed under topical anesthesia and aseptic conditions by the same team of experienced surgeons using the VisuMax femtosecond laser system (Device version 2.10.14; Carl Zeiss Meditec AG, Jena, Germany). The repetition rate and pulse energy were 500 kHz and 130 to 150 nJ, respectively. In all eyes, the intended cap thickness was 110 to 130 μm, the cap diameter was 7.5 to 7.6 mm, and the optical zone of the refractive lenticule was between 6.5 and 6.8 mm. The lenticule was then detached and removed through a 2 mm lateral incision at 120^°^, and postoperative residual stromal bed thickness was at least 280 μm. The surgical technique was the same for all patients and the duration of the procedure was 10 to 15 min. All patients received 0.5% levofloxacin eye drops for 1 week, 4 times a day, and 0.1% fluorometholone eye drops for 4 weeks with weekly tapered doses. No significant complications were observed during postoperative follow-up.

### Statistical analyses

All data were tested for normality using the Kolmogorov-Smirnov method. Friedman test was used to analyze the repeated measurement of the outcome indicators at different time points after surgery, and the post-hoc pairwise comparisons were performed by Wilcoxon signed-rank sum tests with Bonferroni correction. The Mann-Whitney U test was used to compare the postoperative outcome indicators between moderate myopia and high myopia groups. Pearson or Spearman correlation coefficient was used to explore the correlation of postoperative △CV, △Km, △CT, and PTI with preoperative data. Multivariate linear regression was used to analyze the related factors of early postoperative PTI after SMILE, and all variables in the regression model were tested for collinearity by generalized variance inflation factors (VIF), which showed no collinearity was found among the variables (VIF < 2). All statistical analyses were performed using R software (version 4.1.1), and a two-sided *P* value < 0.05 was considered statistically significant.

## Results

### Study population and characteristics

The preoperative refraction was − 4.50 (-5.25, -3.75) and − 7.31 (-7.88, -6.34) in the moderate and high myopia groups, respectively. The two groups were matched for preoperative age, sex, BCVA (logMAR), IOP, Km and CCT (all *P* > 0.05). Patients’ demographics are presented in Table 1.

### CV

The CV of all subjects was different at 1 week, 1 month, 3 months, and 6 months after SMILE (moderate myopia group: *P* = 0.011, high myopia group: *P* = 0.003). The results of the pairwise comparison showed that CV increased at 3 months compared with 1 month (moderate myopia group: *P* = 0.004, high myopia group: *P* = 0.022), but no significant difference in CV between other adjacent time points (all *P* > 0.05). Detailed data of the CV are presented in Table 2. Between the high myopia group and the moderate myopia group, there was no difference in △CV at adjacent time points (all *P* > 0.05).

### Km

As shown in Table 2, the Km of all subjects varied at different time points after SMILE (moderate myopia group: *P* < 0.001, high myopia group: *P* < 0.001). Compared to 1 week postoperatively, there was no significant change in Km at 1 month for moderate myopia (*P* > 0.05), while Km significantly increased for high myopia (*P* < 0.001). Compared to 1 month, Km increased significantly in both groups at 3 months (moderate myopia group: *P* = 0.015; high myopia group: *P* < 0.001). There was no significant difference in Km at 6 months compared to 3 months (all *P* > 0.05). In addition, △Km in the high myopia group was higher than that in the moderate myopia group (1 week to 1 month: *P* = 0.019; 1 month to 3 months: *P* = 0.031), while △KM was not significantly different between the two groups at 3 to 6 months (all *P* > 0.05).

**Table 1:** Preoperative baseline data for the study population

Group	eyes (n)	Age (yrs)	Gender (male/female)	Sphere (D)	Cylinder (D)	SER (D)	BCVA (logMAR)	IOP (mmHg)	Km (D)	CCT (µm)
**Moderate**	60 (30)	24.00 (19.00, 30.00)	18/42	-4.00 (-4.81, -3.50)	-0.75 (-1.25, -0.25)	-4.50 (-5.25, -3.75)	0.00 (0.00, 0.00)	15.00 (14.00, 17.13)	43.40 (42.50, 44.13)	553.00 (538.75, 571.50)
**High**	60 (30)	24.50 (22.00, 30.00)	14/46	-6.75 (-7.25, -5.94)	-0.88 (-1.50, -0.50)	-7.31 (-7.88, -6.34)	0.00 (0.00, 0.00)	17.00 (15.00, 18.00)	43.25 (42.48, 44.00)	560.00 (544.50, 576.25)
***P*** **value**		0.332	0.409	< 0.001	0.004	< 0.001	0.478	0.053	0.615	0.073

M, male; F, female; D, diopters; SER, spherical equivalent refraction; BCVA, best-corrected visual acuity; logMAR, logarithmic minimum angle of resolution; IOP, intraocular pressure; Km, the mean keratometry; CCT, central corneal thickness.

**Table 2:** Postoperative corneal volume and corneal thickness spatial profile after SMILE

CV (mm^3^) / Km (D) / CTSP (µm)	1 wk	1 mo	3 mo	6 mo	*P* value
**CV**					
Moderate myopia	60.40 (59.20, 61.55)	60.40 (58.98, 62.20)	60.55 (59.48, 62.08)^**#**^	60.30 (59.30, 62.33)	**0.011**
High myopia	61.20 (59.68, 62.48)	61.25 (59.60, 62.60)	61.35 (59.68, 62.83)^**#**^	61.50 (60.20, 62.53)	**0.003**
**Km**					
Moderate myopia	39.15 (38.48, 40.43)	39.15 (38.38, 40.70)	39.40 (38.60, 40.73)^*#^	39.30 (38.60, 40.75)^*#^	**< 0.001**
High myopia	37.35 (36.00, 38.40)	37.45 (36.20, 38.70)^*^	37.80 (36.40, 38.73)^*#^	37.85 (36.58, 38.55)^*#^	**< 0.001**
**CTSP**					
**TP**					
Moderate myopia	466.00 (452.25, 484.25)	463.50 (449.75, 485.50)	468.00 (455.75, 489.00)^*#^	469.50 (455.75, 486.00)^*#^	**< 0.001**
High myopia	435.50 (423.75, 459.25)	437.50 (424.75, 461.00)^*^	440.50 (427.00, 460.00)^*#^	442.50 (430.00, 464.50)^*#^	**< 0.001**
**CTR**					
Moderate myopia	468.50 (454.00, 486.25)	465.50 (452.00, 487.00)	469.00 (457.00, 489.25))^*#^	470.00 (458.00, 486.25))^*#^	**< 0.001**
High myopia	437.00 (423.75, 460.00)	439.00 (425.00, 461.25)^*^	441.50 (429.75, 460.50)^*#^	443.00 (432.75, 464.50)^*#^	**< 0.001**
**2 mm**					
Moderate myopia	481.75 (468.81, 500.06)	480.00 (466.31, 503.06)	483.88 (471.88, 502.31)^**#**^	484.25 (471.44, 501.19)^*#^	**< 0.001**
High myopia	455.00 (441.50, 478.75)	456.25 (441.50, 478.44)	460.63 (447.69, 479.56)^*#^	459.63 (448.75, 483.31)^*#^	**< 0.001**
**4 mm**					
Moderate myopia	529.38 (517.31, 554.56)	527.13 (514.25, 552.75)^*^	531.75 (516.63, 552.31)^**#**^	530.88 (516.00, 551.56)^*#^	**< 0.001**
High myopia	517.88 (500.75, 538.00)	518.75 (499.50, 539.63)	520.63 (506.31, 541.25)^*#^	519.38 (505.94, 541.31)^*#^	**< 0.001**
**6 mm**					
Moderate myopia	609.63 (599.25, 637.69)	607.25 (596.25, 637.06)^*^	615.50 (595.94, 638.56)^**#**^	611.63 (600.06, 637.38)^**#**^	**< 0.001**
High myopia	618.75 (598.56, 641.13)	617.25 (596.31, 633.50)	621.25 (600.00, 639.56)^*#^	621.25 (603.13, 642.44)^*#^	**< 0.001**

^*^, Significant difference when compared with 1 week postoperatively (*P* < 0.05); ^#^, Significant difference when compared with 1 month postoperatively (*P* < 0.05); CV, corneal volume; Km, the mean keratometry; CTSP, corneal thickness spatial profile; TP, thinnest point; CTR, center; 2/4/6 mm, ring at 2/4/6 mm diameter.

### CTSP

The CT in different regions of all subjects differed at various time points after SMILE (moderate myopia group: *P* < 0.001, high myopia group: *P* < 0.001). Compared to 1 week, CT at 4, 6 mm rings decreased in the moderate myopia group (*P* = 0.014, 0.005) whereas MCT, CCT increased in the high myopia group (*P* = 0.018, 0.017) at 1 month. Compared to 1 month, CT at the thinnest point, central, and 2, 4, and 6 mm rings showed a statistically significant increase at 3 months in both groups (all *P* < 0.01). However, there was no significant change in CT (TP, CTR, and 2, 4, 6 mm rings) at 6 months compared to 3 months (all *P* > 0.05). Detailed data of the CTSP are shown in Table 2.

**Fig. 1:**
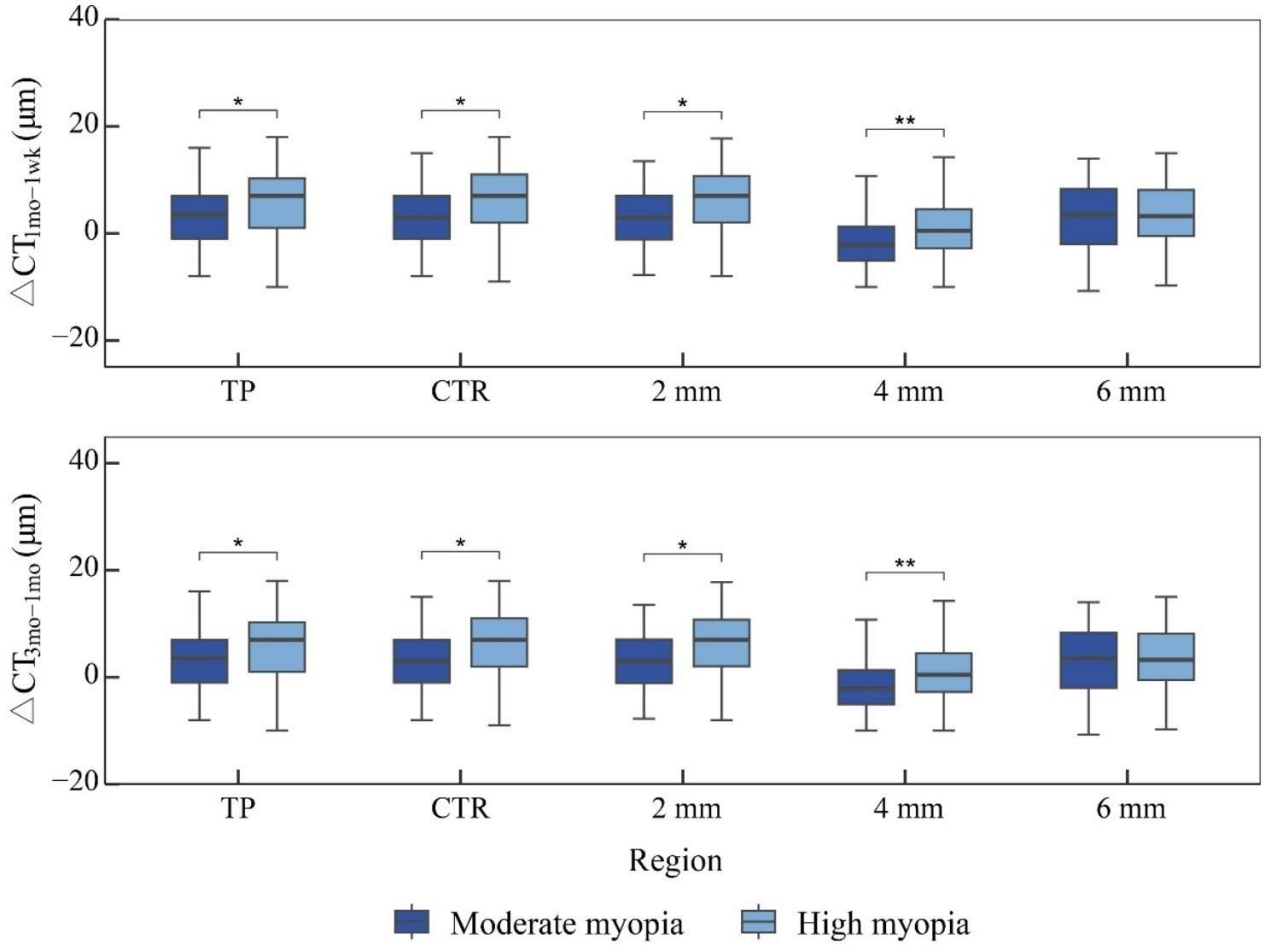
The difference of corneal thickness at adjacent time points in different regions after SMILE TP, thinnest point; CTR, center; 2/4/6 mm, ring at 2/4/6 mm diameter; △CT_1mo − 1wk_, △CT from 1 week to 1 month; △CT_3mo − 1mo_, △CT from 1 month to 3 month; *, *P* < 0.05; **, *P* < 0.01

TP, thinnest point; CTR, center; 2/4/6 mm, ring at 2/4/6 mm diameter; △CT1mo-1wk, △CT from 1 week to 1 month; △CT3mo-1mo, △CT from 1 month to 3 month; *, P < 0.05; **, P < 0.01.

The △CT (1 week to 1 month, 1 month to 3 months) at the thinnest point, central, and 2, 4, and 6 mm rings were higher in the high myopia group than in the moderate myopia group (all *P* < 0.05), but the difference in △CT between the two groups from 3 months to 6 months postoperatively at each region was not significant (all *P* > 0.05) (Fig. 1).

### PTI

The PTI in each region of the study population differed at different time points after SMILE (moderate myopia group: *P* < 0.001, high myopia group: *P* < 0.001). Compared to 1 week, PTI at 2, 4, and 6 mm rings were reduced at 1, 3, and 6 months (moderate myopia group: *P* < 0.05, high myopia group: *P* < 0.05). Between 3 and 6 months, PTI remained decreased at the 2, 4, and 6 mm rings in the high myopia group (all *P* < 0.05), while the moderate myopia group was basically stable (all *P* > 0.05). The PTI of the 2, 4, and 6 mm concentric rings was higher in the high myopia group than in the moderate myopia group at all time points (all *P* < 0.001). Detailed data of the PTI are shown in Table 3.

### Correlation of △CV, △Km, △CT, PTI and preoperative characteristics

**Fig. 2:**
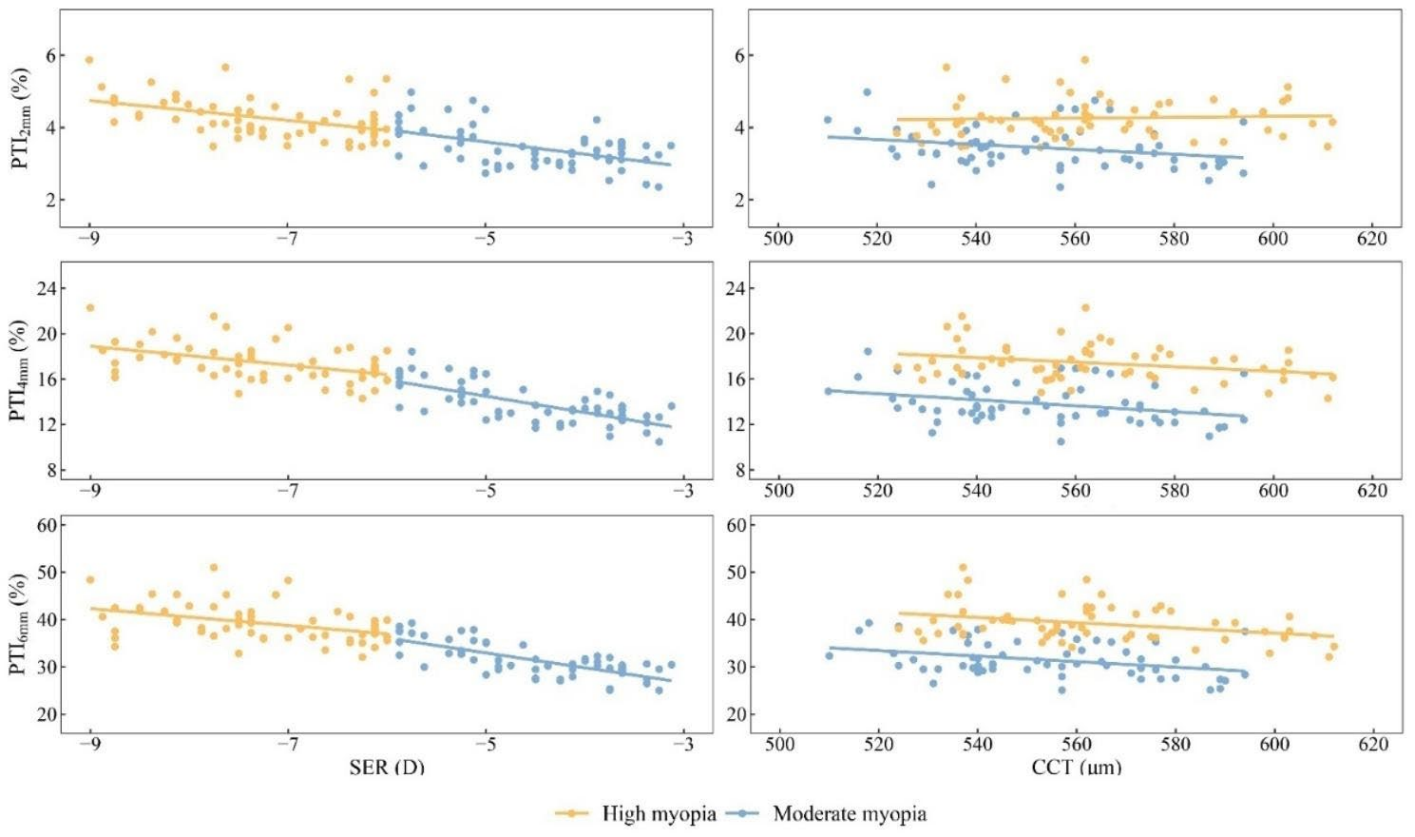
Association of short-term PTI with SER and CCT after SMILE PTI2mm, PTI in a 2 mm diameter region; PTI4mm, PTI in a 4 mm diameter region; PTI6mm, PTI in a 6 mm diameter region; SER, spherical equivalent refraction; CCT, central corneal thickness

Correlations between the amount of change in corneal volume, curvature, and thickness in the early postoperative period and preoperative SER, CCT were not found in all patients (all *P* > 0.05). In the moderate myopia group, PTI in each region in the early postoperative period was negatively correlated with preoperative SER (2 mm: *r* = -0.428, *P* < 0.001; 4 mm: *r* = -0.655, *P* < 0.001; 6 mm: *r* = -0.696, *P* < 0.001), CCT (2 mm: *r* = -0.292, *P* = 0.024; 4 mm: *r* = -0.354, *P* = 0.006; 6 mm: *r* = -0.341, *P* = 0.008), and in the periphery was negatively correlated with age (4 mm: *r* = -0.285, *P* = 0.027; 6 mm: *r* = -0.311, *P* = 0.016), while at the 2 mm concentric ring was not correlated with age (*P* > 0.05). In the high myopia group, there was a negative correlation between postoperative PTI in each region and preoperative SER (2 mm: *r* = -0.447, *P* < 0.001; 4 mm: *r* = -0.451, *P* < 0.001; 6 mm: *r* = -0.440, *P* < 0.001), and at 4, 6 mm rings were negatively correlated with CCT preoperatively (4 mm: *r* = -0.292, *P* = 0.023; 6 mm: *r* = -0.341, *P* = 0.008). However, there was no correlation between PTI and age (all *P* > 0.05) (Fig. 2). Meanwhile, similar results were obtained from multiple linear regression analysis (Table 4).

**Table 3:** The percentage of thickness increase after SMILE

PTI(%)	1 wk	1 mo	3 mo	6 mo	*P* value^a^
**2 mm**					
Moderate myopia	3.58 (3.24, 4.09)	3.57 (3.04, 3.87)^*^	3.32 (3.03, 3.75)^*^	3.36 (3.07, 3.73)^*^	< 0.001
High myopia	4.63 (4.16, 5.06)	4.48 (4.10, 4.75)^*^	4.38 (4.03, 4.75)^*^	4.17 (3.88, 4.59)^*#&^	< 0.001
p value^b^	< 0.001	< 0.001	< 0.001	< 0.001	
**4 mm**					
Moderate myopia	14.29 (12.82, 15.70)	13.83 (12.71, 15.41)^*^	13.49 (12.60, 15.27)^*^	13.36 (12.63, 14.96)^*^	< 0.001
High myopia	18.55 (17.18, 20.04)	17.94 (16.96, 19.29)^*^	17.68 (16.27, 19.20)^*#^	17.07 (16.41, 18.40)^*#&^	< 0.001
p value^b^	< 0.001	< 0.001	< 0.001	< 0.001	
**6 mm**					
Moderate myopia	31.87 (29.81, 34.88)	31.22 (28.94, 34.60)^*^	31.02 (29.09, 34.08)^*^	30.56 (29.43, 33.77)^*^	< 0.001
High myopia	41.38 (38.82, 44.19)	40.48 (37.68, 41.98)^*^	39.45 (36.65, 42.21)^*#^	38.35 (36.51, 41.27)^*#&^	< 0.001
p value^b^	< 0.001	< 0.001	< 0.001	< 0.001	

PTI, the percentage of thickness increase; 2/4/6 mm, ring at 2/4/6 mm diameter; ^*^, Significant difference when compared with postoperative week 1 (*P* < 0.05); ^#^, Significant difference when compared with postoperative month 1 (*P* < 0.05). ^&^, Significant difference when compared with postoperative month 3 (*P* < 0.05). ^a^, Friedman test; ^b^, Mann–Whitney test.

**Table 4:** Influence factors of short-term PTI after SMILE

PTI (%)	Unstandardized coefficients	standardized coefficients	95%CI	*P* value
**Moderate myopia**				
**2 mm**				
Age	-0.015	-0.181	-0.036, 0.005	0.142
Gender	-0.020	-0.017	-0.315, 0.275	0.891
SER	-0.326	-0.488	-0.473, -0.178	**< 0.001**
IOP	-0.005	-0.023	-0.056, 0.045	0.840
CCT	-0.006	-0.242	-0.012, -0.001	**0.039**
**4 mm**				
Age	-0.071	-0.275	-0.117, -0.025	**0.003**
Gender	0.154	0.041	-0.517, 0.824	0.647
SER	-1.373	-0.667	-1.709, -1.037	**< 0.001**
IOP	-0.041	-0.060	-0.156, 0.074	0.480
CCT	-0.023	-0.285	-0.036, -0.009	**0.001**
**6 mm**				
Age	-0.162	-0.304	-0.242, -0.076	**< 0.001**
Gender	1.115	0.144	-0.099, 2.306	0.068
SER	-3.023	-0.714	-3.632, -2.427	**< 0.001**
IOP	-0.012	-0.009	-0.214, 0.198	0.904
CCT	-0.050	-0.301	-0.074, -0.026	**< 0.001**
**High myopia**				
**2 mm**				
Age	0.003	0.029	-0.027, 0.033	0.839
Gender	-0.351	-0.267	-0.704, 0.003	0.051
SER	-0.309	-0.501	-0.473, -0.139	**< 0.001**
IOP	-0.012	-0.055	-0.074, 0.046	0.677
CCT	-0.004	-0.172	-0.011, 0.003	0.233
**4 mm**				
Age	-0.038	-0.122	-0.106, 0.034	0.281
Gender	-0.182	-0.047	-1.008, 0.645	0.659
SER	-1.274	-0.706	-1.662, -0.880	**< 0.001**
IOP	-0.119	-0.180	-0.263, 0.016	0.091
CCT	-0.038	-0.551	-0.053, -0.022	**< 0.001**
**6 mm**				
Age	-0.052	-0.070	-0.216, 0.123	0.540
Gender	0.306	0.034	-1.682, 2.295	0.757
SER	-2.871	-0.674	-3.807, -1.925	**< 0.001**
IOP	-0.236	-0.151	-0.582, 0.089	0.162
CCT	-0.094	-0.581	-0.131, -0.055	**< 0.001**

PTI, the percentage of thickness increase; 2/4/6 mm, ring at 2/4/6 mm diameter; SER, spherical equivalent refraction; IOP, intraocular pressure; CCT, preoperative central corneal thickness.

## Discussion

Since its first clinical application in 2011, the SMILE has shown excellent safety, effectiveness, and predictability in the correction of myopia with or without astigmatism [15–17]. It can scan the corneal stroma evenly, neatly, and accurately through the VisuMax femtosecond laser system, which causes little damage to the cornea and preserves the intact corneal epithelium, Bowman’s membrane, and most of the corneal stroma after surgery, so it can maintain the integrity of corneal morphology and biomechanical stability to a greater extent. Nevertheless, in consideration of safety, the stability of corneal histomorphology is still the focus of attention before and after SMILE. Although there have been previous studies on corneal remodeling after refractive surgery, the changes in corneal spatial profile and PTI at different time points in the early postoperative period have not been comprehensively evaluated. In this study, the CV, CTSP and PTI of patients with moderate and high myopia at 1 week, 1 month, 3 months and 6 months after SMILE were analyzed based on a Pentacam three-dimensional anterior segment analyzer [18, 19] to explore the dynamic changes and possible influencing factors of early postoperative corneal remodeling.

In our study, Between 1 week and 1 month after SMILE, CT in the peripheral region of moderate myopia decreased significantly, while CT in the central region of high myopia increased. △CT in each region of high myopia was higher than that of moderate myopia. The result has not been confirmed in previous studies. The frequency of intraoperative laser scanning, the distance between laser points, and the postoperative medication were essentially the same in all study subjects, so the intraoperative small incision, operation of blunt separation, and postoperative inflammation may be important factors causing corneal thickening [20–22]. Sun et al. [23] studied rabbit corneas after SMILE at multiple time points within 1 month and found that a large number of CD11b-positive cells including monocytes and neutrophils were present at the lateral corneal incision at 1 h after SMILE, which gradually approached the central cornea over time and reached its maximum abundance between 1 day and 1 week postoperatively. IL-1 and TNF-α are produced by corneal epithelial injury after SMILE in patients, and these pro-inflammatory cytokines are released in large quantities, which can enhance corneal inflammatory response and reach a peak within 1 week postoperatively, especially in the central region. Therefore, the rise and fall of CT from 1 week to 1 month after surgery may be related to changes in corneal edema and edema regression caused by inflammatory cascade. Dong et al. [24] observed the histopathology of rabbit corneas after SMILE by light microscopy and found that corneal stromal edema with clearly visible intra-stromal gaps and irregular arrangement of collagen fibers at 1 week, stromal edema subsided and collagen fibers were regularly arranged at 1 month, and intra-stromal gaps disappeared and the structure of each layer stabilized at 3 months. The results of transmission electron microscopy also showed that the mitochondria of keratinocytes were swollen and cell energy turnover was impaired within 1 month, and that returned to normal after 3 months. Compared with the moderate myopia group, the lens made by the high myopia group was thicker, and the stromal edema reaction of the cornea was more obvious within 1 month after surgery, especially in the central region, and took longer time for the corneal edema to subside. In this study, CV and CT at each region increased in both groups from 1 month to 3 months after SMILE, then stabilized after 3 months. In addition, the ΔCT in all regions was higher in the high myopia group than in the moderate myopia group, and the Km of the two groups of subjects showed a consistent trend. This conclusion is in line with those of earlier studies [9, 25]. Wei et al. [26] found that mucoid secretions adhered to the corneal stromal space after intraoperative lens removal and collected mainly in the central corneal region, increasing CV and CT after SMILE. Other studies have shown that activated fibroblasts in the corneal stroma after SMILE increase the synthesis and activity of the extracellular matrix, such as the increasing activity of fibronectin [27–29], which can further promote the migration of fibroblasts and thus complete the proliferation and remodeling of the corneal stroma, that may also be an important factor for the increase of CV and CT after SMILE. Moreover, previous studies on corneal epithelial thickness after SMILE found that the anterior corneal surface sagged and the corneal stroma showed morphological and structural changes due to lens removal, which induced the corneal epithelium to form a regular and smooth corneal surface through the compensatory mechanism to maintain normal optical function, so that the corneal epithelium is continuously hyperplasia and remodeling like a convex lens. The reconstructive process tended to be stable at 3 months postoperatively [30], which could explain a rebound in Km and an increase in CCT within 3 months after SMILE. At the same time, the compensation mechanism was more significant in patients with high myopia and high astigmatism [31, 32], thus the changes in Km and CT are more obvious in the high myopia group compared to the moderate myopia group.

The differences in corneal remodeling after surgery between moderate and high myopia are also shown in the PTI outcome. In our study, we found that PTI at 2, 4, and 6 mm stabilized after 1 month postoperatively in patients with moderate myopia, while it continued to change between 3 and 6 months in the high myopia group. Hence, patients with high myopia took longer to stabilize after SMILE, and had higher PTI compared to the moderate myopia group at all postoperative time points. Lazaridis et al. [9] reported that PTI at 2 mm ring remained stable at 2 months postoperatively, while it continued to decrease at 4, 6, and 8 mm rings, which is similar to our study. In general, after the lenticule is extracted, the arc length of the back of the corneal cap is longer than that of the residual stroma, and the difference between arc lengths contributes to a corrugated contact surface, meanwhile, the collagen fibers are rearranged, all of which have an impact on the stability of the corneal [33]. After SMILE, compared to the moderate myopia group, the patients with high myopia had thicker removed lenticule, greater differences in arc length, which resulted in a more uneven contact surface potentially between the corneal cap and the residual stroma. In addition, the more volume of Bowman’s membrane and corneal stroma was damaged postoperatively, CCT decreased more than preoperatively, and the rearrangement of collagen fibers is enhanced [34], which had a greater impact on postoperative corneal stability. Hence, the high myopia group reached the stability of corneal thickness and morphology later than the moderate myopia group after SMILE.

After further exploring the association of postoperative corneal volume, curvature, and thickness changes with preoperative characteristics in both groups, we found that in addition to higher refraction, the increased peripheral corneal PTI in the early postoperative period may also be associated with thin CCT and low age before operation. Some studies have shown that thin preoperative CCT and insufficient postoperative residual corneal stroma have an effect on the biomechanical properties of the cornea, resulting in weaker postoperative corneal tensile strength [35–37]. The thicker the cornea, the tighter the connections of collagen fibers and proteoglycans in the stroma, and the less change in postoperative corneal thickness and morphology. The negative correlation between age and PTI can be explained by two points. On the one hand, the biomechanical response of corneal stroma to microlens removal is correlated with age [38, 39]. With the increase of age, the number, diameter, non-enzymatic reaction and carbonylation reaction of corneal collagen fibers increase, the space between collagen fibers and the number of glycosaminoglycans in the extracellular matrix decreases, leading to the reduction of corneal viscosity and the increase of hardness, which is associated with postoperative corneal remodeling and changes. On the other hand, the basement membrane nerve plexus of the corneal epithelium plays a nutritional and sustaining role in the regularity of the corneal surface and in the proliferation and integrity of the epithelium, which has an important effect on corneal healing after SMILE [40]. Metabolomics studies have shown that the increase in age is often accompanied by the accumulation of uric acid and the decrease of metabolic markers such as ascorbic acid, taurine, glutamine and alanine [41–43], which adversely affects the ability of corneal nerve fibers to maintain and regenerate [44]. Meanwhile, Tummanapalli et al. [45] found that corneal nerve fiber density and corneal nerve fractal dimension decreased with the increase of age. Therefore, older people have less change in corneal thickness and better corneal stability after SMILE than younger people. However, our study only found an association between age and PTI in the moderate myopia group, which may be due to the small study sample size and the large effect of refraction on PTI.

There are some limitations of our study. We only analyzed the early postoperative corneal remodeling process, while CV, CTSP, and PTI may continue to change from 6 months to 1 year after SMILE, and longer-term follow-up is needed for the exploration of postoperative corneal spatial remodeling. Furthermore, the sample size of this study was relatively small, and future studies with larger samples are still needed to investigate corneal remodeling after refractive surgery.

## Conclusions

Our study evaluated the trends of CV, Km, CTSP and PTI in people with moderate and high myopia within 6 months after SMILE, to further understand the process and rule of healing response of corneal tissue after surgery in people with different refractive levels. The corneal remodeling and duration after SMILE were different in patients with moderate and high myopia. Meanwhile, the dynamic changes of the cornea tended to be stable after 3 months, and the early postoperative changes in corneal remodeling at various regions may be related to the preoperative SER, CCT and age, which can provide a specific reference for the assessment of preoperative indications, the design of surgical plan and early postoperative follow-up. In summary, the remodeling, repair and stabilization of the cornea after refractive surgery is a dynamic and long-term process that still needs to be followed by histopathological studies and long-term observation.

### Electronic supplementary material

Below is the link to the electronic supplementary material.

Supplementary Material 1

## Data Availability

The datasets used and analyzed during the current study are available from the corresponding author (Ting Shen& Chaoyang Hong) upon reasonable request.

## Abbreviations

SMILE: Small incision lenticule extraction
CT: Corneal thickness
CV: Corneal volume
CTSP: Corneal thickness spatial profile
Km: Mean keratometry
PTI: Percentage of thickness increase
SER: Spherical equivalent refraction
CCT: Central corneal thickness
BCVA: Best-corrected visual acuity
IOP: Intraocular pressure
TP: Thinnest point
MCT: Minimum corneal thickness
CTR: Central
ΔCV: The change in corneal volume
ΔCT: The change in corneal thickness
VIF: Variance inflation factor
logMAR: Logarithm of the minimal angle of resolution
