# Supplementary figures and images for: Corneal remodeling after SMILE for moderate and high myopia: short-term assessment of spatial changes in corneal volume and thickness
Source: BMC Ophthalmol. 2023 Oct 6;23:402. doi: 10.1186/s12886-023-03148-0 (PMC10559442; doi:10.1186/s12886-023-03148-0)

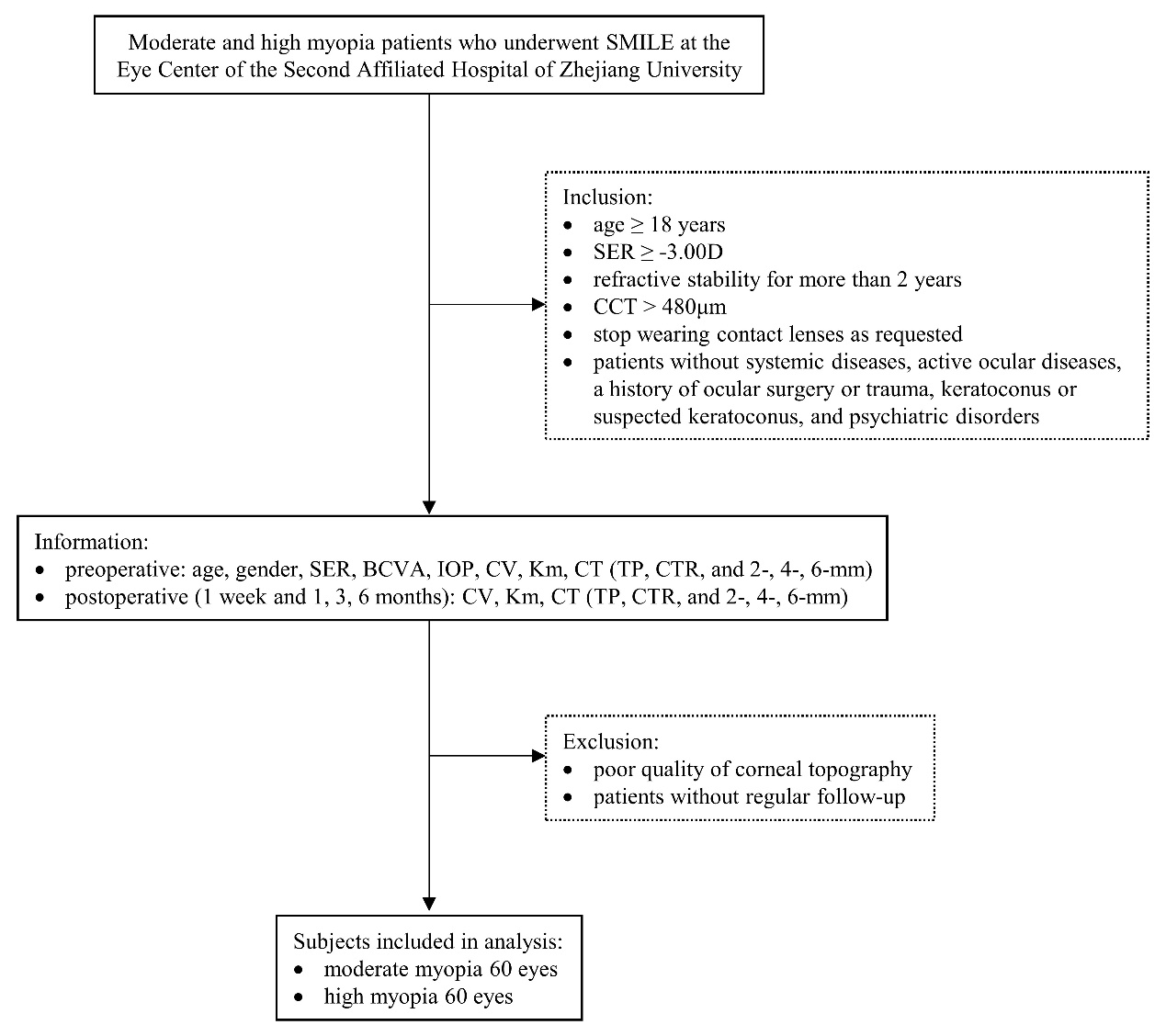


**Supplementary Figure 1.** Flowchart for information collection

Supplement: Supplementary file 1 — Supplementary Material 1 [file 12886_2023_3148_MOESM1_ESM.docx]
